# Supplementary material for: Impact of Capsid and Genomic Integrity Tests on Norovirus Extraction Recovery Rates
Source: Foods. 2023 Feb 15;12(4):826. doi: 10.3390/foods12040826 (PMC9957022; doi:10.3390/foods12040826)
Supplement: Supplementary file 1 [file foods-12-00826-s001.zip › Supplementary Figure S1 Flow charts.pdf]

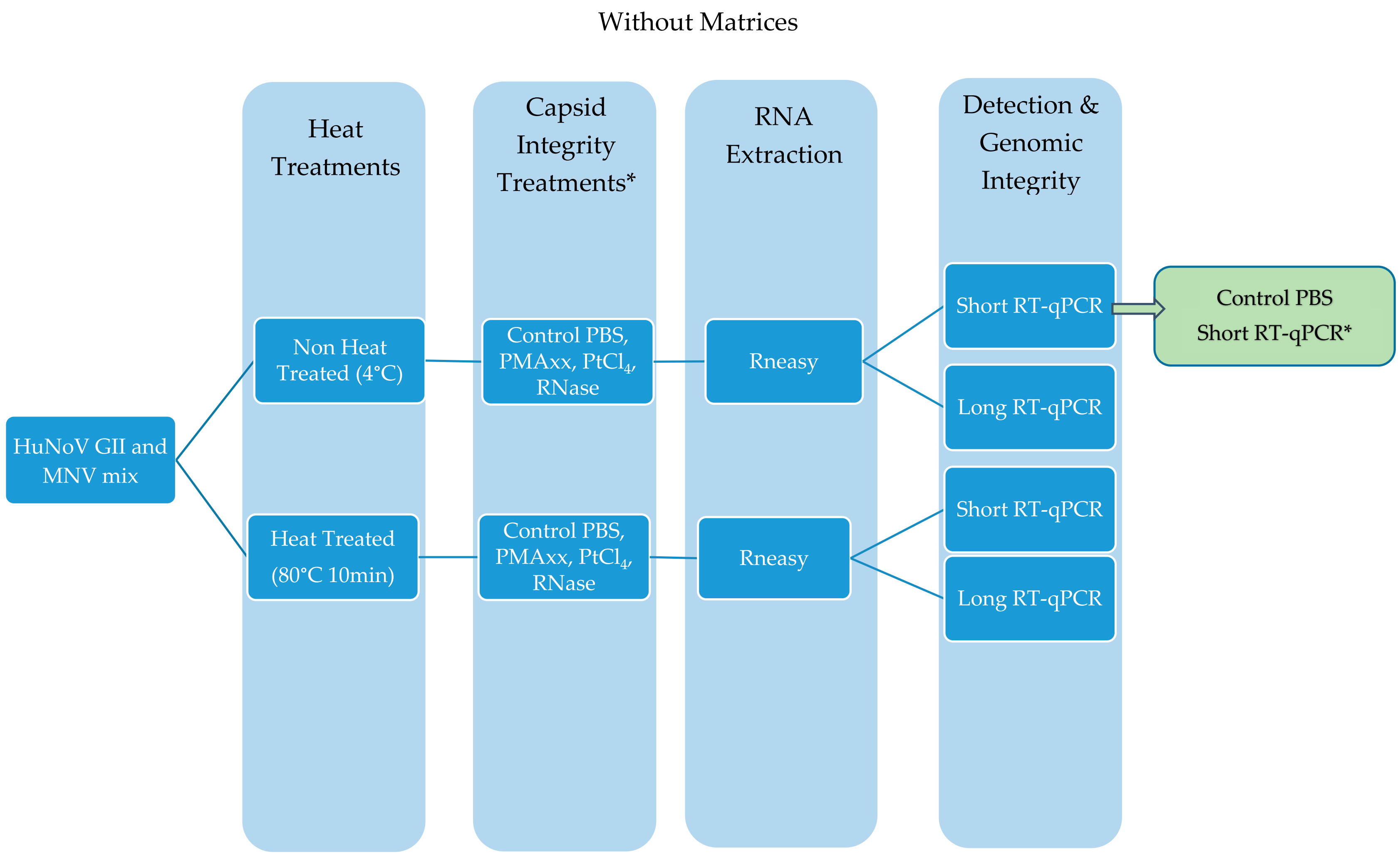

(a)

Supplementary Figure S1: Experiment flow chart without matrices(a). All results were compared to the Control PBS Short RT-qPCR recovery results. *The Control PBS Short RT-qPCR recovery results are equivalent to the Inoculum.

With Matrices

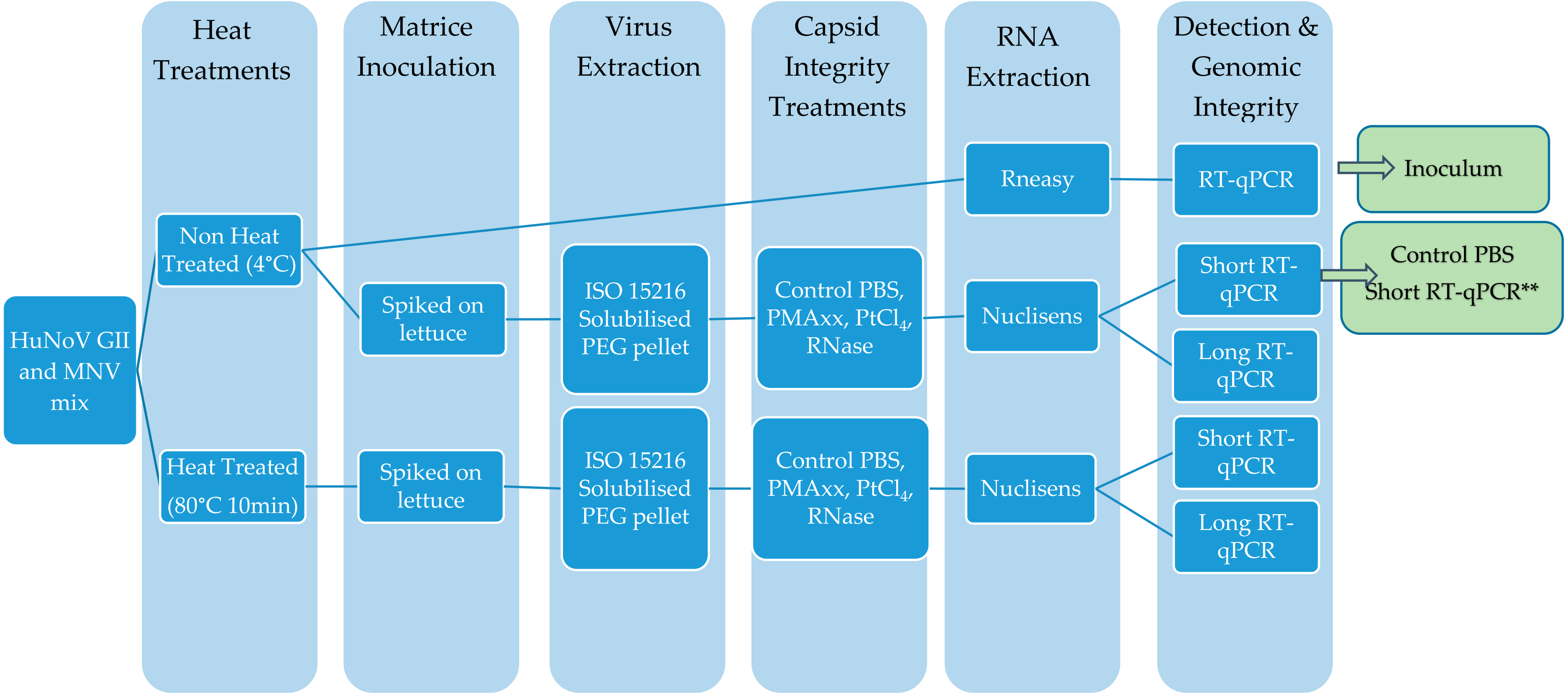

(b)

Supplementary Figure S1: Experiment flow chart with matrices(b). All results were compared to the Control PBS Short RT-qPCR recovery results. **The Control PBS Short RT-qPCR recovery results are not equivalent to the Inoculum since the virus was spiked on matrices..
