# Supplementary material for: Impact of Capsid and Genomic Integrity Tests on Norovirus Extraction Recovery Rates
Source: Foods. 2023 Feb 15;12(4):826. doi: 10.3390/foods12040826 (PMC9957022; doi:10.3390/foods12040826)
Supplement: Supplementary file 1 [file foods-12-00826-s001.zip › Supplementary Materials Table S1 Primers probes and RNA transcripts used in this study.pdf]

Supplementary Table S1: Primers, probes and RNA transcripts used in this study.

Methods	Primer, Probe, Transcript	Sequence 5'-3'	Reference
		Norovirus GII	
RT-qPCR, qPCR	QNIF2d	ATG TTC AGR TGG ATG AGR TTC TCW GA	Loisy et al. (2005)
RT-qPCR, qPCR	FAM-QNIFS-BHQ-1	AGC ACG TGG GAG GGC GAT CG	Loisy et al. (2005)
RT-qPCR, qPCR	COG2R	TCG ACG CCA TCT TCA TTC ACA	Kageyama et al. (2003)
RT-qPCR, RT long vs short ratio	HuNov GII transcript	GGGCGAATTGGGTACGATCGATGCGGCCTCGA ATTCATGTTCAGATGGATGAGATTCTCAGATCT GAGCACGTGGGAGGGCGATCGCAATCTGGCTC CCAGTACTGAGGGTTGTTTGTGAATGAAGATGGCGTCGAA	Raymond et al. (2021)
		Murine norovirus	
RT-qPCR, qPCR	FW-ORF1/ORF2	CAC GCC ACC GAT CTG TTC TG	Baert et al. (2008)
RT-qPCR, qPCR	RV-ORF1/ORF2	GCG CTG CGC CAT CAC TC	Baert et al. (2008)
RT-qPCR, qPCR	FAM- ORF1/ORF2-MGBNFQ	CGC TTT GGA ACA ATG	Baert et al. (2008)
RT-qPCR, RT long vs short ratio	MNV transcript	CACGCCACCGATCTGTTCTGCGCTGGGTGCGCTTTGGA ACAATGGATGCTGAGACCactgagggttgcgttagacggCCGCAGG AACGCTCAGCAGTCTTTGTGAATGAGGATGAGTGATGGCGCAGCGC	Raymond et al. (2021)
		Long-range RT	
RT	Tx30SxN	GAC TAG TTC TAG ATC GCG AGC GGC CGC CCT TTT TTT TTT TTT TTT TTT TTT TTT T	Raymond et al. (2022)
