# Supplementary material for: Impact of Capsid and Genomic Integrity Tests on Norovirus Extraction Recovery Rates
Source: Foods. 2023 Feb 15;12(4):826. doi: 10.3390/foods12040826 (PMC9957022; doi:10.3390/foods12040826)
Supplement: Supplementary file 1 [file foods-12-00826-s001.zip › Supplementary Materials Table S2 Impact of Different Capsid Integrity Treatments without matrices.pdf]

Supplementary Table S2
Impact of Different Capsid Integrity Treatments on the Recovery of Heat-Treated HuNoV and MNV without matrices.

Virus	Detection	Integrity Treatment	4°C		80°C	
			Positive/Tested	Log Reduction vs short RT-qPCR Control PBS	Positive/Tested	Log Reduction vs short RT-qPCR Control PBS
HuNoV	Short RT-qPCR	Control PBS	55/55		36/36	
		PMaxx	6/6	0.01	6/6	2.71
		$PtCl_4$	11/11	0.19	7/12	2.90
		RNase	18/18	0.01	12/12	1.50
	Long RT-qPCR	Control PBS	66/66	0.01	27/36	0.53
		PMaxx	6/6	0.12	0/6	>3
		$PtCl_4$	20/20	0.20	0/21	>3
		RNase	9/9	0.01	6/9	2.47
MNV	Short RT-qPCR	Control PBS	70/70		36/36	
		PMaxx	6/6	0.64	3/6	2.86
		$PtCl_4$	21/21	0.58	11/12	2.48
		RNase	24/24	0.23	12/12	1.06
	Long RT-qPCR	Control PBS	60/60	0.01	33/33	0.43
		PMaxx	6/6	0.42	0/6	>3
		$PtCl_4$	18/18	0.38	5/12	2.01
		RNase	9/9	0.10	9/9	0.84
