# Supplementary material for: Impact of Capsid and Genomic Integrity Tests on Norovirus Extraction Recovery Rates
Source: Foods. 2023 Feb 15;12(4):826. doi: 10.3390/foods12040826 (PMC9957022; doi:10.3390/foods12040826)
Supplement: Supplementary file 1 [file foods-12-00826-s001.zip › Supplementary Materials Table S3 Impact of Different Capsid Integrity Treatments with matrices.pdf]

Supplementary Table S3

Impact of Different Capsid Integrity Treatments on the Recovery of Heat-Treated HuNoV and MNV Spiked on Fresh Lettuce, Using the ISO 15216-1:2017 Methodology.

Virus	Detection	Integrity Treatment	4ºC		80ºC	
			Positive/Tested	Log Reduction vs short RT-qPCR Control PBS	Positive/Tested	Log Reduction vs short RT-qPCR Control PBS
HuNoV	Short RT-qPCR	Control PBS	12/12		17/17	
		PMaxx	6/6	0.04	0/6	>3
		$PtCl_4$	6/6	0.27	6/6	0.51
		RNase	12/12	0.04	17/17	1.55
	Long RT-qPCR	Control PBS	12/12	0.09	15/17	0.96
		PMaxx	6/6	0.39	1/6	1.28
		$PtCl_4$	6/6	0.16	1/6	2.46
		RNase	12/12	0.06	8/17	1.86
MNV	Short RT-qPCR	Control PBS	12/12		17/17	
		PMaxx	6/6	0.53	1/6	2.85
		$PtCl_4$	6/6	0.24	6/6	0.20
		RNase	12/12	0.28	17/17	1.38
	Long RT-qPCR	Control PBS	12/12	-0.18	14/17	0.54
		PMaxx	6/6	0.74	2/6	2.33
		$PtCl_4$	6/6	-0.24	6/6	0.5
		RNase	12/12	-0.07	6/17	1.08
