# Supplementary figures and images for: Impact of Capsid and Genomic Integrity Tests on Norovirus Extraction Recovery Rates
Source: Foods. 2023 Feb 15;12(4):826. doi: 10.3390/foods12040826 (PMC9957022; doi:10.3390/foods12040826)

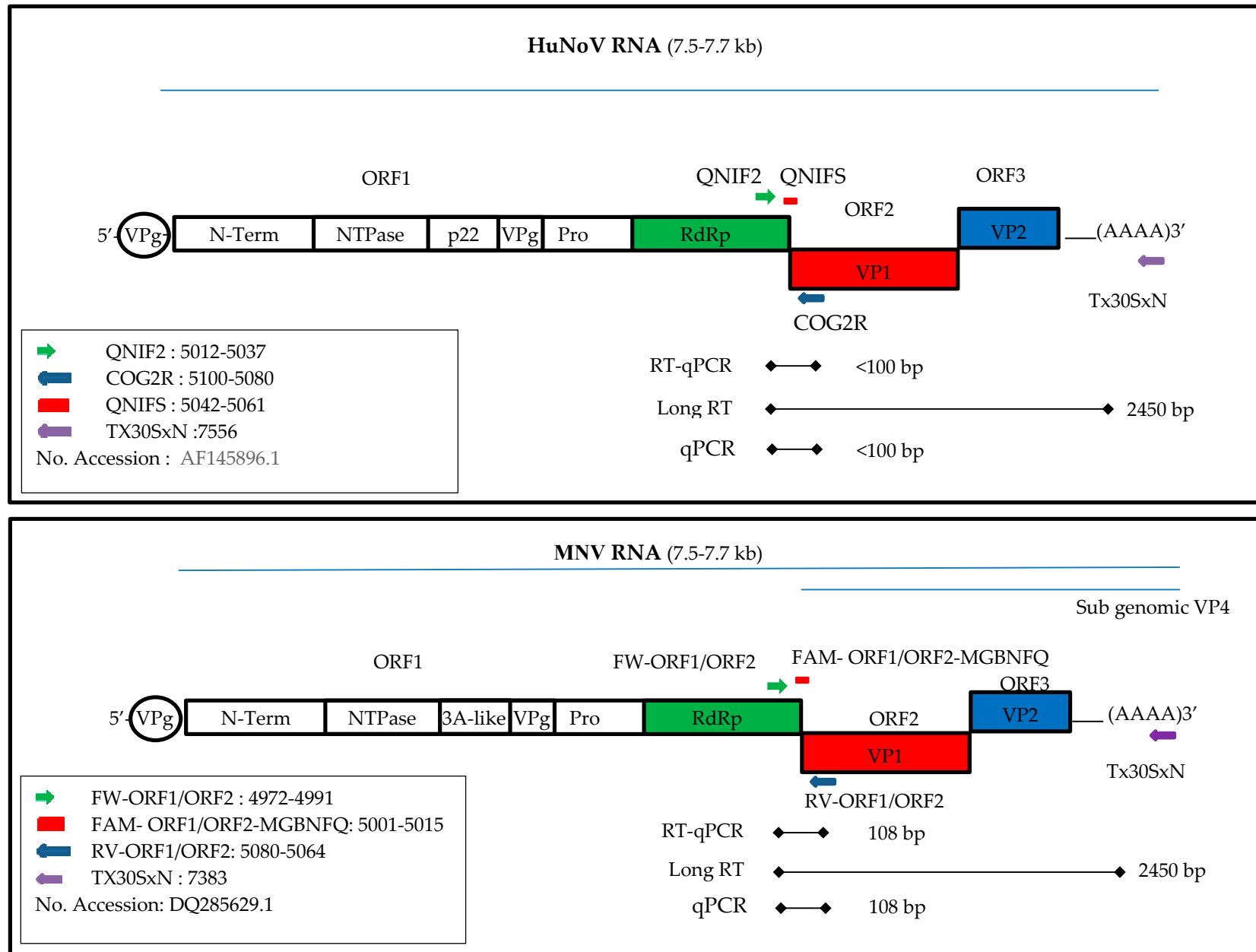

**Supplementary Figure S2: Primer and probe locations.**

Supplement: Supplementary file 1 [file foods-12-00826-s001.zip › Supplementary Figure S2 Primer and probe locations.pdf]
